# The non-antibiotic macrolide EM900 inhibits rhinovirus infection and cytokine production in human airway epithelial cells

**DOI:** 10.14814/phy2.12557

**Published:** 2015-10-13

**Authors:** Nadine Lusamba Kalonji, Kazuhiro Nomura, Tetsuaki Kawase, Chiharu Ota, Hiroshi Kubo, Takeya Sato, Teruyuki Yanagisawa, Toshiaki Sunazuka, Satoshi Ōmura, Mutsuo Yamaya

**Affiliations:** 1 Department of Advanced Preventive Medicine for Infectious Disease, Tohoku University Graduate School of MedicineSendai, Japan; 2 Department of Molecular Pharmacology, Tohoku University Graduate School of MedicineSendai, Japan; 3 Department of Otolaryngology-Head and Neck Surgery, Tohoku University Graduate School of MedicineSendai, Japan; 4 Laboratory of Rehabilitative Auditory Science, Tohoku University Graduate School of Biomedical EngineeringSendai, Japan; 5 Kitasato Institute for Life Sciences, Kitasato UniversityTokyo, Japan; 6 Graduate School of Infection Control Sciences, Kitasato UniversityTokyo, Japan

**Keywords:** Airway epithelial cell, COPD, EM900, inflammation, rhinovirus

## Abstract

The anti-inflammatory effects of macrolides may be associated with a reduced frequency of exacerbation of chronic obstructive pulmonary disease (COPD). However, because the long-term use of antibiotics may promote the growth of drug-resistant bacteria, the development of a treatment to prevent COPD exacerbation with macrolides that do not exert anti-bacterial effects is necessary. Additionally, the inhibitory effects of nonantibiotic macrolides on the replication of rhinovirus (RV), which is the major cause of COPD exacerbation, have not been demonstrated. Primary cultures of human tracheal epithelial cells and nasal epithelial cells were pretreated with the nonantibiotic macrolide EM900 for 72 h prior to infection with a major group RV type 14 rhinovirus (RV14) and were further treated with EM900 after infection. Treatment with EM900 before and after infection reduced RV14 titers in the supernatants and viral RNA within the cells. Moreover, cytokine levels, including interleukin (IL)-1*β* and IL-6, were reduced in the supernatants following RV14 infection. Treatment with EM900 before and after infection also reduced the mRNA and protein expression of intercellular adhesion molecule-1 (ICAM-1), which is the receptor for RV14, after infection and reduced the activation of the nuclear factor kappa-B protein p50 in nuclear extracts after infection. Pretreatment with EM900 reduced the number and fluorescence intensity of the acidic endosomes through which RV RNA enters the cytoplasm. Thus, pretreatment with EM900 may inhibit RV infection by reducing the ICAM-1 levels and acidic endosomes and thus modulate the airway inflammation associated with RV infections.

## Introduction

Rhinoviruses (RVs) are the major cause of exacerbation of chronic obstructive pulmonary disease (COPD) (Seemungal et al. 2000). In addition to a variety of drugs, such as long-acting muscarinic antagonists, long-acting *β*_2_-agonists and inhaled corticosteroids, macrolide antibiotics, including erythromycin, clarithromycin, and azithromycin, also reduce the frequency of COPD exacerbation (Suzuki et al. 2001b; Seemungal et al. 2008; Yamaya et al. 2008; Albert et al. 2011). However, the long-term use of antibiotics may promote the growth of drug-resistant bacteria. Therefore, the development of a treatment to prevent COPD exacerbation with macrolides that do not exert antibacterial effects is necessary (Ni et al. 2015).

The major group of RVs enters the cytoplasm of infected cells after binding to the receptor known as intercellular adhesion molecule (ICAM)-1 (Greve et al. 1989; Casasnovas and Springer 1994). The entry of the RNA of this RV group into the cytoplasm of infected cells has been suggested to be mediated by destabilization due to receptor binding and endosomal acidification (Perez and Carrasco 1993; Casasnovas and Springer 1994). The macrolide antibiotics bafilomycin and erythromycin inhibit infection by this group of RVs by reducing ICAM-1 expression and increasing the endosomal pH (Suzuki et al. 2001a, 2002). The inhibitory effects of erythromycin on RV infection and the production of inflammatory cytokines (Suzuki et al. 2002) may be associated with the clinical benefits of macrolides for the prevention of COPD exacerbation. The novel 12-membered nonantibiotic macrolide EM900 (Sugawara et al. 2011) has been shown to reduce the levels of inflammatory cytokines such as interleukin (IL)-1*β* and tumor necrosis factor (TNF)-*α* in human airway epithelial cells (Otsu et al. 2011). However, the inhibitory effects of EM900 on RV infection are unclear.

We studied the effects of the novel 12-membered nonantibiotic macrolide EM900 on RV infection in primary cultures of human tracheal epithelial (HTE) cells and human nasal epithelial (HNE) cells.

## Materials and Methods

### Human tracheal and nasal epithelial cell cultures

The HTE cells were derived from the tracheae of 27 patients after death (age: 67 ± 3 years, mean ± SEM; 12 females and 15 males). The HNE cells were obtained from excised nasal specimens from the coronoid processes of subjects undergoing endoscopic endonasal sinus surgery (*n* = 27; age: 62 ± 4 years; 13 females and 14 males). The HTE and HNE cells were isolated and cultured as previously described (Suzuki et al. 2001a; Yamaya et al. 2014b). Briefly, for the HTE and HNE cell cultures**,** surface epithelial cells of the human tracheal and nasal mucosa were isolated by exposure to 0.05% protease and cultured in plastic tubes with round bottoms (16 mm in diameter and 125 mm in length; Becton Dickinson, Franklin Lakes, NJ) or on coverslips in Petri dishes coated with human placental collagen as previously described (Suzuki et al. 2001a; Yamaya et al. 2014b).

The cells were plated at a density of 5 × 10^5^ viable cells/mL in plastic tubes. The plastic tubes were fixed in an inclined stainless-steel tube rack (30 cm wide, 10 cm high and 10 cm deep, TE-HER TUBE RACK INCLINABLE® RF-6; Hirasawa Works Co. Ltd., Tokyo, Japan) and placed in a humid incubator. The tubes were kept stationary, and the cells were immersed in 1 mL of Dulbecco’s Modified Eagle’s medium (DMEM)–Ham’s F-12 medium (50/50, vol/vol) containing 2% ultroser G and cultured at 37°C in an atmosphere of 5% CO_2_–95% air in the incubator.

None of the patients was being treated with macrolides at the time of death or surgery. This study was approved by the Tohoku University Ethics Committee.

### Human embryonic fibroblast cell culture

Human embryonic fibroblast (HEF) cells (HFL-III cells, Riken Bio Resource Center Cell Bank, Cell No: RCB0523; Tsukuba, Japan) were cultured as previously described (Yamaya et al. 2014b).

### Viral stock

The RV14 (a major group RV) stock was prepared from a patient with a common cold by infecting HEF cells as previously described (Yamaya et al. 2014b).

### Detection and titration of viruses

RV14 in the supernatants (cell culture medium) was detected and titrated using HEF cells with endpoint methods by infecting replicate confluent HEF cells in plastic 96-well plates (Becton Dickinson) with serial 10-fold dilutions of virus-containing supernatants as previously described (Suzuki et al. 2001a; Yamaya et al. 2014b). Virus-containing supernatants were added to the replicate HEF cells in the wells (200 *μ*L/well) of the 96-well plates. HEF cells in the wells were cultured at 33°C in 5% CO_2_–95% air, and the presence of the typical cytopathic effects of rhinovirus was observed in all replicate cells for 7 days (168 h) as previously described (Suzuki et al. 2001a; Yamaya et al. 2014b). The rates of RV14 release into the supernatants are expressed as the tissue culture infective dose (TCID_50_) units/mL (Suzuki et al. 2001a; Yamaya et al. 2014b).

### Quantification of rhinoviral RNA

To quantify the RV14 RNA content and *β*-actin mRNA expression levels in the epithelial cells, a two-step real-time quantitative reverse transcription (RT)-PCR assay with the TaqMan technique was performed using the TaqMan® Gene Expression Master Mix (Applied Biosystems, Bedford, CA) as previously described (Yamaya et al. 2014b). An RT-PCR for *β*-actin was also performed as previously described (Yamaya et al. 2014b). The expression of the RV RNA was normalized to the constitutive expression of the *β*-actin mRNA.

### Viral infection of epithelial cells

Infection of the HTE and HNE cells with RV14 was performed using previously described methods (Yamaya et al. 2014b). A stock solution of RV14 (100 *μ*L in each tube, 1.0 × 10^4^ TCID_50_ units/100 *μ*L, 2.0 × 10^−2^ TCID_50_ units/cell) was added to the HTE and HNE cells in the tubes. After a 1-h incubation at 33°C in 5% CO_2_–95% air, the viral solution was removed and the epithelial cells were rinsed once with 1 mL of phosphate-buffered saline (PBS). Then, the cells were fed with 1 mL of fresh medium containing 2% USG. The opening of the tubes was sealed with rubber plugs, and the cells in the tubes were cultured at 33°C by rolling in an incubator.

### Pretreatment with EM900

HTE and HNE cells were pretreated with either EM900 (10 μmol/L unless otherwise specified) or vehicle (0.05% dimethyl sulfoxide [DMSO]) for 3 days (72 h) prior to infection to examine the effects of EM900 on RV14 release, RV14 RNA replication, ICAM-1 expression, endosomal acidification, cytokine secretion, and NF-*κ*B activation; alternatively, the cells were cultured in medium alone. To examine the effects of EM900 on cell functions other than endosomal acidification, the cells were also treated with EM900 or vehicle or cultured in medium alone after RV14 infection until the end of the experimental period.

### Collection of supernatants for measurement

To examine the effects of EM900 on viral release, the supernatants were collected at 1 day (24 h), 3 days (72 h), and 5 days (120 h) after infection. In preliminary experiments, the secretion of IL-1*β* and IL-6 into the supernatants increased at 72 h after infection. Therefore, to examine the effects of EM900 on the secretion of IL-1*β* and IL-6, the supernatants were collected before infection and at 72 h after infection.

### Effects of EM900 on susceptibility to rhinovirus infection

The effects of EM900 on the susceptibility to RV14 infection were evaluated as previously described (Suzuki et al. 2001a). The HTE and HNE cells were pretreated with EM900 (10 μmol/L) or vehicle for 72 h before infection with RV14 until the time of infection or cultured in medium alone for 72 h. The epithelial cells were exposed to serial 10-fold dilutions of RV14 at doses ranging from 10^1^ to 10^5^ TCID_50_ units/mL in media containing EM900, vehicle or neither for 1 h at 33°C. After exposure to RV14, the cells were rinsed with PBS, and fresh medium without the addition of EM900 or vehicle was added. The cells in the tubes were cultured at 33°C by rolling in an incubator.

Supernatants were collected at 24 h, 72 h, and 120 h after RV14 infection with the methods described above (*Collection of supernatants for measurements*). Because we found in preliminary experiments that the supernatants collected at 72 h after infection contained the maximum viral titers, we measured the RV14 titers in the supernatants collected at 72 h after infection with the HEF cell assay described above to assess whether infection occurred at each dose (10^1^, 10^2^, 10^3^, 10^4^ or 10^5^ TCID_50_ units/mL) of RV14 used. After the addition of virus-containing supernatants to the HEF cells, the cells were observed for the presence of the typical cytopathic effects of rhinovirus infection for 7 days (168 h) (Suzuki et al. 2001a).

### Measurement of ICAM-1 expression and cytokine production

ICAM-1 mRNA levels were examined via two-step real-time RT-PCR analysis using the methods described above (*Quantification of rhinoviral RNA*) with a forward primer designed for ICAM-1 (Yamaya et al. 2014b). The concentration of the soluble form of ICAM-1 (sICAM-1) and the levels of IL-1*β* and IL-6 in the supernatants were examined using specific enzyme-linked immunosorbent assays (ELISAs).

### Measurement of changes in the acidic endosomes

The distribution and fluorescence intensity of the acidic endosomes in the cells were measured with LysoSensor DND-189 dye (Molecular Probes, Eugene, OR) as previously described (Suzuki et al. 2001a) using live-cell imaging. Cells on coverslips in Petri dishes were observed with a fluorescence microscope (OLYMPUS IX70; OLYMPUS Co. Ltd., Tokyo, Japan). The excitation wavelength was 443 nm, and the emitted light from the cells was detected through a 505-nm filter. The fluorescence intensity was calculated using a fluorescence image analyzer system (Lumina Vision®; Mitani Co. Ltd., Fukui, Japan) equipped with a fluorescence microscope. HTE and HNE cells were pretreated with EM900 or vehicle or cultured in medium alone. The fluorescence intensity of the acidic endosomes was measured in 100 cells, and the mean value of the fluorescence intensity was expressed as a percentage of the control value compared with the fluorescence intensity of the cells prior to any treatment.

We studied the effects of a long period of pretreatment with EM900 (10 μmol/L, 72 h) on acidic endosomes because the cells were pretreated with EM900 for 72 h prior to RV14 infection.

### NF-kappa B assay

The presence of the p50 and p65 subunits in the nuclear extracts was assessed using a TransFactor Family Colorimetric Kit-NF-*κ*B (BD Bioscience/CLONTECH) according to the manufacturer’s instructions, as previously described (Yamaya et al. 2014b). To examine the effects of EM900 on the NF-*κ*B subunits in the cells before RV14 infection, the cells in the tubes were pretreated with EM900 or vehicle or cultured in medium alone for 72 h prior to the extraction of nuclear proteins. To examine the effects of EM900 on NF-*κ*B after RV14 infection, the cells were pretreated with EM900 or vehicle or cultured in medium alone prior to the infection, and cultured in medium containing EM900 or vehicle or in medium alone at 33°C by rolling in an incubator for 72 h after infection; then, the nuclear proteins were extracted from the cells.

### Measurement of airway epithelial cell damage

To examine the HTE and HNE cell damage, the number of floating cells in the supernatants, which were detached from the cell sheets adhered on the culture vessels of plastic tubes, and the number and viability of the adhered cells were measured by trypan blue exclusion (Yamaya et al. 2014a). Lactate dehydrogenase (LDH) concentrations in the supernatants were also measured (Yamaya et al. 2014a).

### Statistical analysis

The results are expressed as the mean ± SEM. For the comparison of data between the two groups, Student’s *t*-test or the Mann-Whitney *U*-test was performed. For the comparison of data between more than two groups, statistical analysis was performed using analysis of variance (ANOVA) and subsequent post hoc analysis using Bonferroni’s method. For all analyses, values of *P *<* *0.05 were considered significant. The number of donors from whom the cultured epithelial cells were obtained is referred to as n.

## Results

### Effects on rhinovirus replication in epithelial cells

**Figure 1:**
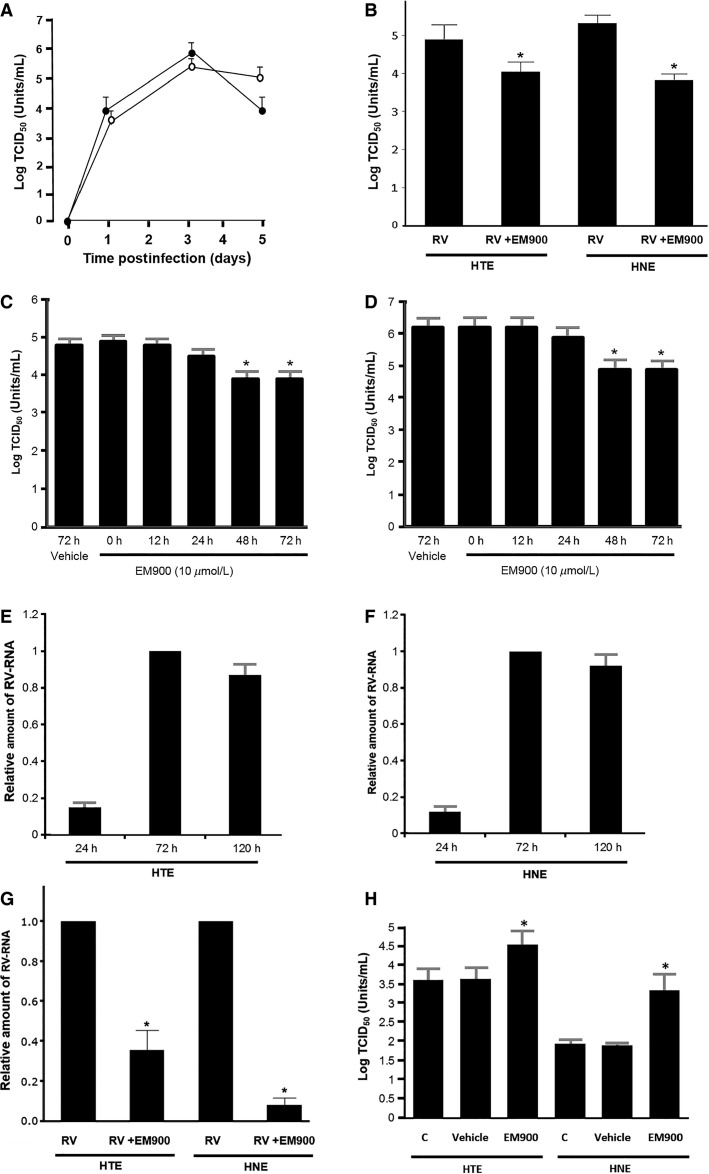
(A) The time course of viral release into the supernatants of human tracheal epithelial (HTE) cells (open circles) and human nasal epithelial (HNE) cells (closed circles) cultured in medium alone obtained at different time points after RV14 infection. (B) Viral release into the supernatants of the HTE and HNE cells collected at 72 h after infection following pretreatment with EM900 (RV + EM900) or vehicle (RV). (A and B) The results are presented as the mean ± SEM for six samples. Significant differences compared to the pretreatment with vehicle (RV) are indicated by *P <* *0.05. (C and D) Time course of the effects of EM900 (10 μmol/L) on viral release into the supernatants collected at 72 h after infection from HTE cells (C) or HNE cells (D) pre treated for time periods ranging from 0 h to 72 h and the viral release from cells pretreated with vehicle for 72 h. The results are the mean ± SEM from five samples. Significant differences compared to cells cultured in medium alone (time 0) are indicated by *P <* *0.05. Viral titers in the supernatants of the untreated cells (time 0) did not differ from the titers in the cells pretreated with vehicle (vehicle for 72 h). (E and F) The time course of RV14 RNA production in the untreated HTE (E) and HNE (F) cells cultured in medium alone obtained at different time points after RV14 infection. RV14 RNA expression was normalized to the constitutive expression of *β*-actin mRNA. RV14 RNA expression at 72 h was set to 1.0. The results are expressed as the relative amount of RNA expression (ratio) compared with the levels of RV14 RNA expression at 72 h and are reported as the mean ± SEM from three samples. (G) Replication of RV14 RNA in HTE cells and HNE cells at 72 h after infection following pretreatment with EM900 (RV + EM900) or vehicle (RV). RV14 RNA expression was normalized to the constitutive expression of *β*-actin mRNA. RV14 RNA expression in the cells pretreated with vehicle (RV) was set to 1.0. The results are expressed as the relative amount of RNA expression (ratio) compared with the maximum levels of RV14 RNA expression at 72 h in cells pretreated with vehicle and are reported as the mean ± SEM for three (trachea) or four (nasal epithelia) samples. Significant differences compared to treatment with the vehicle alone (RV) are indicated by *P <* *0.05. (H) The minimum dose of RV14 necessary to cause infection in HTE and HNE cells pre treated with EM900 or vehicle (Vehicle) or in the untreated cells cultured in medium alone (Control; C). The results are presented as the mean ± SEM for three samples. Significant differences compared to pretreatment with the vehicle alone (Vehicle) are indicated by *P <* *0.05.

RV14 was detected in the supernatants at 24 h. The levels of the viral titer in the supernatants increased significantly over time for the first 3 days (72 h), and consistent viral titer levels were detected at 5 days (120 h) after infection (Fig.1A).

Pretreatment of the HTE and HNE cells with EM900 significantly decreased the RV14 viral titers in the supernatants at 72 h compared with the titers in the cells pretreated with vehicle (0.05% DMSO) (Fig.1B). EM900 pretreatment for 48 h or longer prior to infection significantly decreased the RV14 viral titers in the HTE and HNE cells (Fig.1C and D). RV14 viral titers in the supernatants of the HTE and HNE cells cultured in medium alone at 72 h after infection did not differ from the RV14 viral titers in the cells pretreated with vehicle (Fig.1C and D), showing that the EM900 vehicle did not affect the RV14 titers.

Pretreatment with EM900 reduced RV14 release into the supernatants of HTE and HNE cells in a concentration-dependent manner at concentrations of 1 μmol/L or greater (5.9 ± 0.2 vs. 5.4 ± 0.1 log TCID_50_ units/mL in HTE cells at 1 μmol/L, *n* = 3 and 6.2 ± 0.3 vs. 5.5 ± 0.2 log TCID_50_ units/mL in HNE cells at 1 μmol/L, *n* = 3) (*P *<* *0.05).

We also tested possible cytotoxicity due to EM900 in HTE and HNE cells cultured in plastic tubes (Yamaya et al. 2014a). Pretreating the HTE cells with EM900 or vehicle for 72 h did not increase the proportion of dead cells among the attached cells (97 ± 1% in EM900, 98 ± 1% in the vehicle, and 97 ± 1% in the medium alone, *n* = 4, *P *>* *0.20). Similarly, the number of detached HTE cells in the supernatants from the tubes pretreated with EM900 or vehicle for 72 h did not differ from the number of detached cells in the tubes cultured in medium alone (0.71 ± 0.14 × 10^4^ in EM900, 0.70 ± 0.13 × 10^4^ in vehicle, and 0.72 ± 0.14 × 10^4^ in the medium, number/tube, *n* = 4, *P *>* *0.20). Furthermore, pretreatment with EM900 or the vehicle did not increase the LDH concentration in the supernatants (33 ± 3 U/L in EM900, 30 ± 3 U/L in vehicle, and 31 ± 4 in the medium alone, *n* = 3, *P *>* *0.20).

Similarly, pretreating the HNE cells with EM900 or vehicle for 72 h did not increase the proportion of dead cells among the attached cells (98 ± 1% in EM900, 97 ± 1% in vehicle, and 98 ± 1% in the medium alone, *n* = 4, *P *>* *0.20). Likewise, the number of detached HNE cells in the supernatants from the tubes pretreated with EM900 or vehicle for 72 h did not differ from the number of detached cells in the tubes cultured in the medium alone (0.72 ± 0.15 × 10^4^ in EM900, 0.73 ± 0.14 × 10^4^ in vehicle, and 0.74 ± 0.14 × 10^4^ in the medium, number/tube, *n* = 4, *P *>* *0.20). Finally, pretreatment with EM900 or vehicle did not increase the LDH concentration in the supernatants (29 ± 2 U/L in EM900, 28 ± 3 U/L in vehicle, and 28 ± 2 in the medium alone, *n* = 3, *P *>* *0.20).

### Effects on viral RNA

RV14 RNA was consistently observed in the HTE and HNE cells beginning 24 h after infection. Subsequent increases were observed between 24 and 72 h after infection, and the maximum levels of RV14 RNA were observed at 72 h after infection (Fig.1E and F), which was in agreement with previous reports in HTE cells (Suzuki et al.; Yamaya et al. 2014b 2001a).

Pretreatment of the cells with EM900 significantly decreased the RV14 RNA levels in the HTE and HNE cells after infection (Fig.1G). In contrast, RV14 RNA levels in the HTE and HNE cells pretreated with vehicle did not differ from the RV14 RNA levels in the untreated cells cultured in medium alone at 72 h after infection (relative amount of RV RNA compared with the untreated cells: 1.04 ± 0.08 in the HTE cells and 1.03 ± 0.06 in the HNE cells, *n* = 3, *P *>* *0.30).

We used the cycle threshold (Ct) values to assess the amount of RV RNA using the RT-PCR method; the amount of RV RNA was normalized to the amount of *β*-actin, which was used as a housekeeping gene in the present study. Therefore, to examine whether RV14 infection influenced *β*-actin mRNA expression, we assessed the Ct values of *β*-actin mRNA before and after infection. In the untreated HTE cells, the Ct values of *β*-actin mRNA after infection (23.6 ± 0.5, *n* = 4, *P *>* *0.3) did not differ from the values before infection (24.0 ± 0.7, *n* = 4). Similarly, in the HNE cells, the Ct values of *β*-actin mRNA after infection (22.7 ± 0.9, *n* = 3, *P *>* *0.1) did not differ from the values before infection (24.1 ± 0.3, *n* = 3).

### Effects on susceptibility to rhinovirus infection

The minimum dose of RV14 that was necessary to cause infection in the cells pretreated with vehicle of EM900 did not differ from the dose required for the untreated cells (Fig.1H). Therefore, pretreatment of the cells with vehicle did not alter the susceptibility of the HTE and HNE cells to RV14 infection. In contrast, pretreatment of the cells with EM900 significantly decreased the susceptibility of the HTE and HNE cells to RV14 infection. The minimum dose of RV14 that was necessary to cause infection in the cells pre treated with EM900 was significantly higher than the dose required for the cells pre treated with vehicle (Fig.1H).

### Effects on ICAM-1 expression

**Figure 2:**
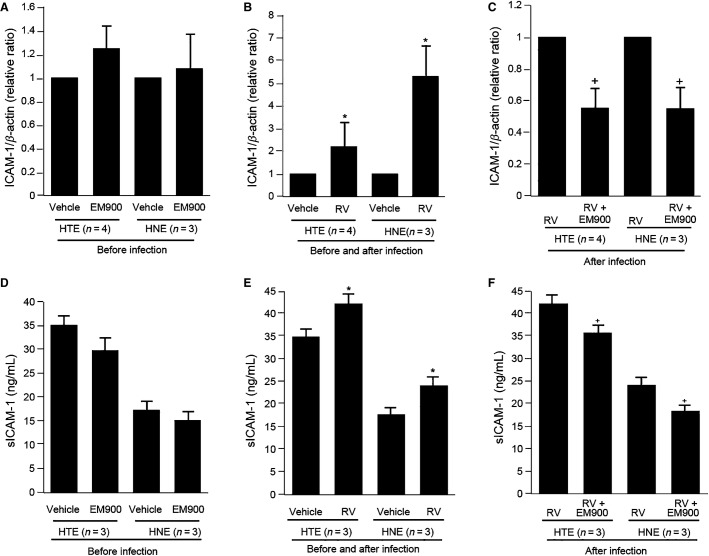
(A and D) The expression of ICAM-1 mRNA (A) and the sICAM-1 concentration in the supernatants (D) of HTE and HNE cells pretreated with EM900 or vehicle before RV14 infection. (B and E) The expression of ICAM-1 mRNA (B) and sICAM-1 concentration (E) at 72 h after RV14 infection (RV) or in the absence of the infection (Vehicle) in cells pretreated with vehicle. Significant differences compared to pretreatment with vehicle (Vehcle) in the absence of infection are indicated by *P <* *0.05. (C and F) The expression of ICAM-1 mRNA (C) and the sICAM-1 concentration (F) at 72 h after RV14 infection in cells pre treated with EM900 (RV + EM900) or vehicle (RV). Significant differences from the cells pretreated with vehicle after infection (RV) are indicated by ^+^*P *<* *0.05. (A–C) The expression of ICAM-1 mRNA was normalized to the *β*-actin mRNA. The expression of ICAM-1 mRNA in cells pretreated with vehicle in the absence of infection (A and B) and in cells pretreated with vehicle after infection (C) was set to 1.0.

Pretreatment of the cells with EM900 did not reduce baseline ICAM-1 mRNA expression by the HTE or HNE cells compared with the levels detected in the cells pretreated with vehicle prior to RV14 infection (Fig.2A). The levels of ICAM-1 mRNA expression increased following RV14 infection (Fig.2B). In contrast, pretreatment and treatment with EM900 before and after RV14 infection reduced ICAM-1 mRNA expression after infection (Fig.2C).

ICAM-1 mRNA expression in the cells pretreated with vehicle did not differ from the mRNA expression in the untreated cells prior to infection (the relative amount of ICAM-1 mRNA compared with the untreated cells: 1.02 ± 0.07 in the HTE cells and 1.04 ± 0.08 in the HNE cells, *n* = 3, *P *>* *0.30). Thus, pretreatment of the cells with vehicle did not affect ICAM-1 mRNA expression prior to infection.

Similarly, pretreatment and treatment of the cells before and after infection with vehicle of EM900 did not change ICAM-1 mRNA expression after infection. ICAM-1 mRNA expression at 72 h after infection in the cells pretreated and treated with vehicle before and after infection did not differ from the mRNA expression in the untreated cells (the relative amount of ICAM-1 mRNA at 72 h after infection compared with that in the untreated cells: 1.05 ± 0.02 in the HTE cells and 1.03 ± 0.05 in the HNE cells, *n* = 3, *P *>* *0.30).

Pretreatment of the cells with EM900 did not reduce the baseline sICAM-1 concentration in the HTE or HNE cells compared with the levels in the cells pretreated with vehicle prior to RV14 infection (Fig.2D) (*P *>* *0.10). In contrast, the sICAM-1 concentration was increased 72 h after RV14 infection (Fig.2E). When the cells were pretreated and treated with EM900 before and after infection, the sICAM-1 concentration in the HTE and HNE cells was reduced after RV14 infection (Fig.2F).

The concentrations of sICAM-1 in the untreated cells (34.4 ± 2.5 ng/mL in the HTE cells, and 16.9 ± 1.8 ng/mL in the HNE cells, *n* = 3, *P *>* *0.10) did not differ from the concentrations in the cells pretreated with vehicle prior to infection. Similarly, the concentrations of sICAM-1 in the untreated cells (42.0 ± 2.3 ng/mL in the HTE cells and 23.9 ± 1.6 ng/mL in the HNE cells, *n* = 3, *P *>* *0.10) did not differ at 72 h after infection from the concentrations in the cells pretreated with vehicle.

### Effects on cytokine production

**Figure 3:**
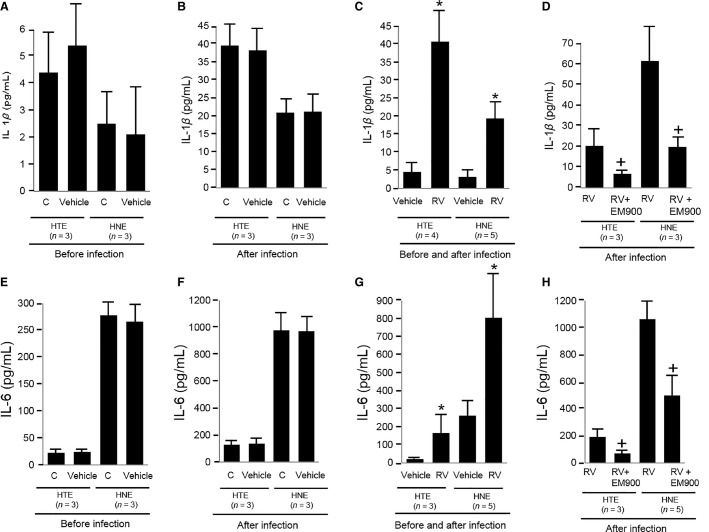
(A, B and E, F) The secretion of IL-1*β* (A and B) and IL-6 (E and F) into the supernatants of HTE and HNE cells pretreated with vehicle of EM900 (Vehicle) or cultured in medium alone (untreated cells) (Control; C) prior to infection (A and E) and at 72 h after infection (B and F). The results are given as the mean ± SEM. (C and G) The release of IL-1*β* (C) and IL-6 (G) into the supernatants at 72 h after RV14 infection (RV) or in the absence of the infection (Vehicle) in HTE and HNE cells pre treated with vehicle. The results are given as the mean ± SEM. Significant differences compared to pretreatment with the vehicle in the absence of the infection (Vehicle) are indicated by *P <* *0.05. (D and H) The release of IL-1*β* (D) and IL-6 (H) into the supernatants of HTE and HNE cells at 72 h after RV14 infection following pretreatment with EM900 (RV + EM900) or vehicle (RV). The results are given as the mean ± SEM. Significant differences compared to pretreatment with vehicle after infection (RV) are indicated by ^+^*P *<* *0.05.

The secretion of IL-1*β* by untreated HTE and HNE cells prior to infection did not differ from the concentration in the cells pretreated with vehicle of EM900 (Fig.3A). At 72 h after infection, the secretion of IL-1*β* in the untreated cells also did not differ from the concentration in the cells pretreated with vehicle (Fig.3B). RV14 infection increased the secretion of IL-1*β* in the supernatants of the untreated cells (Fig.3A and B) and the cells pretreated with vehicle (Fig.3A–C) at 72 h after infection. In contrast, when the cells were pretreated with EM900 before infection and treated with EM900 after infection, the infection-induced secretion of IL-1*β* at 72 h after infection was reduced compared with the vehicle-treated cells (Fig.3D).

Similarly, the secretion of IL-6 by the untreated cells prior to infection did not differ from the concentration in the cells pre treated with vehicle (Fig.3E). At 72 h after infection, the secretion of IL-6 by the untreated cells also did not differ from the secretion by the cells treated with vehicle (Fig.3F). RV14 infection increased the secretion of IL-6 into the supernatants of the untreated HTE and HNE cells (Fig.3E and F) and the cells pretreated with vehicle (Fig.3E–G) at 72 h after infection. In contrast, when the cells were pretreated with EM900 before infection and treated with EM900 after infection, the infection-induced secretion of IL-6 at 72 h after infection was reduced compared with vehicle-treated cells (Fig.3H).

### Effects on the acidification of endosomes

**Figure 4:**
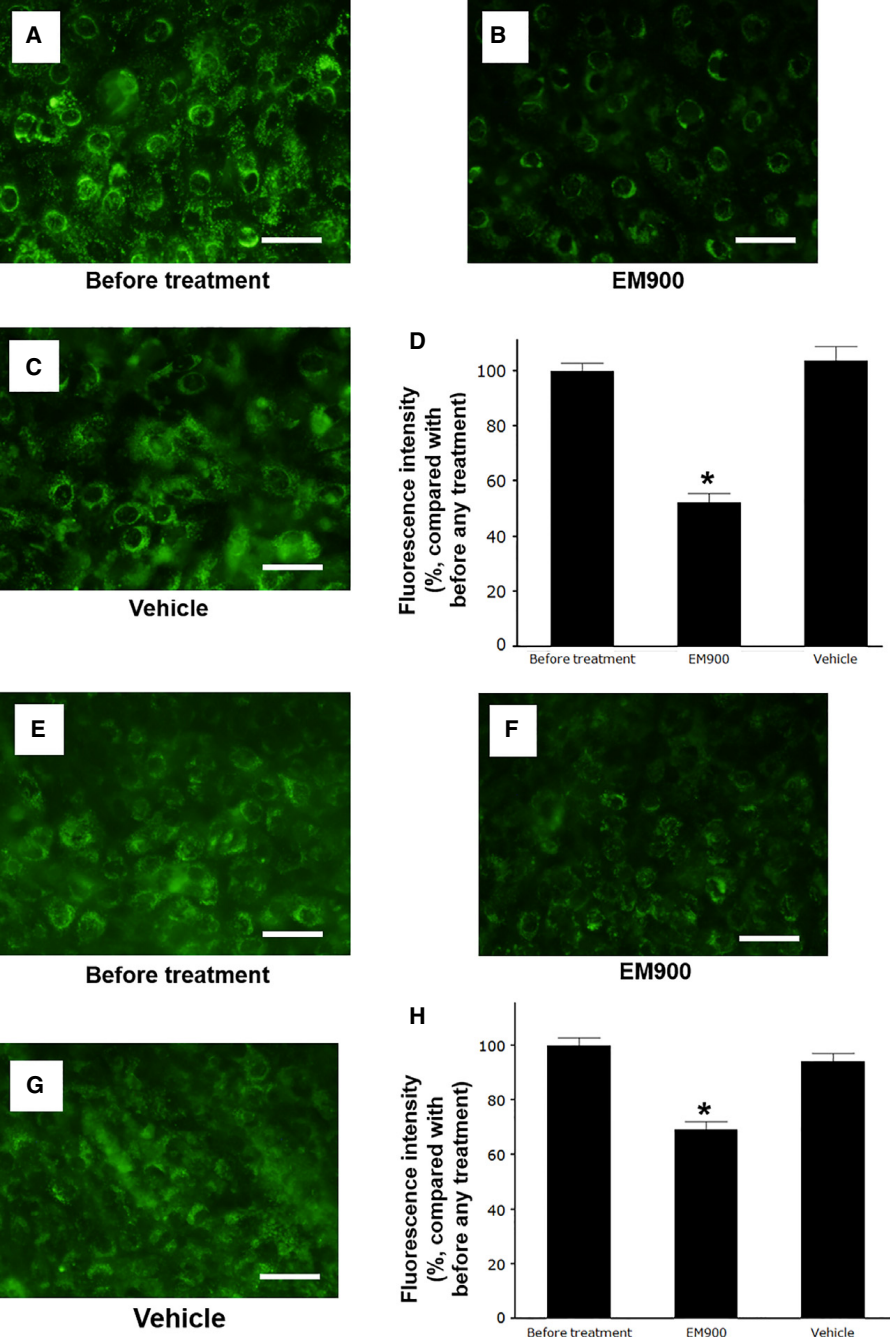
(A–C and E–G) Changes in the distribution of acidic endosomes exhibiting green fluorescence in the HTE (A–C) and HNE (E–G) cells at 72 h after pre treatment with EM900 (B and F) or vehicle (C and G) or untreated cells cultured in medium alone (before treatment) (A and E) (Bar = 100 *μ*m). (D and H) The effects of pretreatment with EM900 or vehicle assessed in HTE cells (D) and HNE cells (H) at 72 h after treatment or in untreated cells (before treatment) on the fluorescence intensity of acidic endosomes. The results are expressed as the mean ± SEM for five tracheae. Significant differences compared to the vehicle alone (Vehicle) are indicated by *P <* *0.05.

Pretreatment with vehicle for 72 h did not alter the number of acidic endosomes in the HTE cells (Fig.4A and C) or HNE cells (Fig.4E and G) as determined by the presence of green fluorescence and the fluorescence intensity of the acidic endosomes (Fig.4D and H) compared with the intensity in cells prior to any treatment. In contrast, pretreatment with EM900 reduced the number of acidic endosomes in the HTE cells (Fig.4B) and HNE cells (Fig.4F). Moreover, pretreatment with EM900 reduced the fluorescence intensity (Fig.4D and H) compared to cells that were pretreated with vehicle and cells prior to any treatment.

### Effects on NF-kappa B

**Figure 5:**
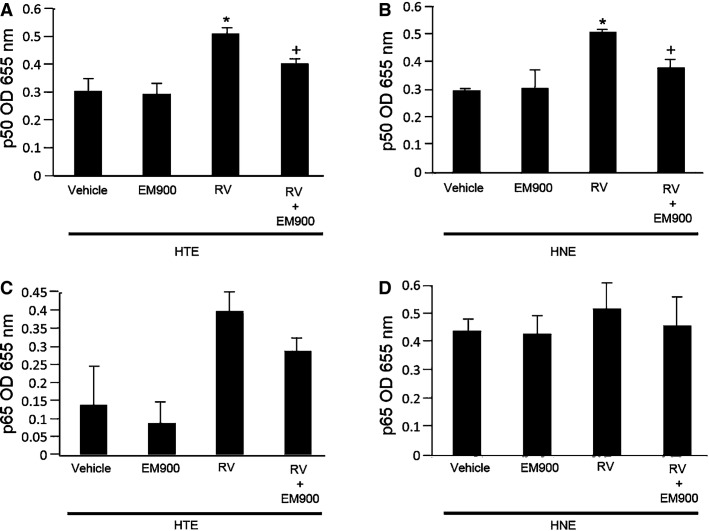
Amount of p50 (A and B) and p65 (C and D) in the nuclear extracts of the HTE (A and C) and HNE cells (B and D) before and at 72 h after RV14 infection. The cells were pretreated with EM900 or vehicle (Vehicle) for 72 h prior to infection or pretreated starting at 72 h before infection and treated until 72 h after infection with EM900 (RV + EM900) or vehicle (RV). The results are expressed as the optical density (OD) and are given as the mean ± SEM for four (HTE cells) or three (HNE cells) samples. Significant differences compared to the vehicle alone (Vehicle) before infection are indicated by *P <* *0.05. Significant differences compared to pretreatment with vehicle after infection (RV) are indicated by ^+^*P *<* *0.05.

Pretreatment with EM900 did not reduce the levels of NF-*κ*B p50 in the nuclear extracts compared with HTE and HNE cells pretreated with vehicle prior to RV14 infection (Fig.5A and B). RV14 infection increased the amount of NF-*κ*B p50 in both the HTE and HNE cells pretreated with vehicle, and EM900 reduced the amount of p50 following infection when the cells were pretreated with EM900 prior to infection and treated with EM900 after infection (Fig.5A and B).

Pretreatment with EM900 did not reduce the levels of NF-*κ*B p65 compared with HTE and HNE cells pretreated with vehicle prior to RV14 infection (Fig.5C and D). RV14 infection tended to increase the amount of NF-*κ*B p65 in both the HTE and HNE cells and pretreatment with EM900 tended to reduce the amount of p65 after infection, although these reductions did not reach statistical significance (Figs5C and D).

The amount of NF-*κ*B p50 and p65 in the untreated HTE cells (0.32 ± 0.03, *n* = 4, and 0.14 ± 0.05, *n* = 3, respectively) and the untreated HNE cells (0.31 ± 0.02, *n* = 4, and 0.46 ± 0.06, *n* = 3) prior to RV14 infection did not differ from the cells pretreated with vehicle (*P *>* *0.02). Similarly, the amount of NF-*κ*B p50 and p65 in the untreated HTE cells (0.52 ± 0.02, *n* = 4, and 0.42 ± 0.04, *n* = 3, respectively) and the untreated HNE cells (0.51 ± 0.01, *n* = 4, and 0.52 ± 0.05, *n* = 3) at 72 h after infection did not differ from the cells pretreated with vehicle (*P *>* *0.02).

## Discussion

In this study, we demonstrated that pretreatment with the nonantibiotic macrolide EM900 reduced RV14 titers and cytokine concentrations in the supernatants of primary cultures of HTE and HNE cells and RV14 RNA replication within the cells. Pretreatment with EM900 reduced the mRNA and protein expression of ICAM-1, the receptor for the major group of RVs (Greve et al. 1989), after RV14 infection. Pretreatment with EM900 also reduced the number and fluorescence intensity of acidic endosomes through which RV RNA enters the cytoplasm (Perez and Carrasco 1993; Casasnovas and Springer 1994) and increased the minimum dose of RV14 that was necessary to cause a viral infection. These findings suggest that EM900 inhibited RV14 infection partially by reducing the production of its receptor and the number of acidic endosomes.

Macrolides reduce the frequency of COPD exacerbation (Suzuki et al. 2001b; Seemungal et al. 2008; Yamaya et al. 2008; Albert et al. 2011). Long-term treatment with erythromycin did not increase the number of erythromycin-resistant bacteria in the sputum (Seemungal et al. 2008). However, the long-term use of an antibiotic may promote the growth of drug-resistant bacteria, and thus the development of a treatment to prevent COPD exacerbation with nonantibiotic macrolides is necessary (Ni et al. 2015). Sugawara et al. (Sugawara et al. 2011) developed the 12-membered erythromycin analogue EM900 and demonstrated that EM900 did not exert antibacterial activities against several representative organisms, including *Staphylococcus aureus*, *Escherichia coli,* and *Klebsiella pneumoniae*. These findings suggest that EM900 does not exhibit any antibacterial activity.

A variety of mediators, including IL-6 and IL-8, are related to airway inflammation during COPD exacerbation caused by RV infections (Seemungal et al. 2000). EM900 has anti-inflammatory and immunomodulatory activities (Sugawara et al. 2011, 2012), and nonantibiotic macrolides in the EM900 series (EM905 and EM914) reduce inflammation during acid-induced colitis in rats (Sugawara et al. 2012). Otsu et al. demonstrated that EM900 and erythromycin reduced IL-8 production in the human type II lung cell line A549 (Otsu et al. 2011). In the current study, pretreatment with EM900 reduced the RV14 infection-induced secretion of IL-1*β* and IL-6. These inhibitory effects are consistent with the results of previous studies showing that the macrolides bafilomycin and erythromycin reduce the RV infection-induced production of IL-1*β* and IL-6 in HTE cells (Suzuki et al. 2001a, 2002). ICAM-1 also plays a vital role in the airway inflammation of patients with COPD (Riise et al. 1994). The inhibitory effects of EM900 on ICAM-1 expression observed in this study are consistent with the findings of previous studies that macrolides bafilomycin and erythromycin reduce ICAM-1 expression after RV14 infection in HTE cells (Suzuki et al. 2001a, 2002). Similar to erythromycin, EM900 may exert inhibitory effects on RV infection-induced airway inflammation.

In the present study, pretreatment of epithelial cells with EM900 reduced ICAM-1 expression and the production of pro-inflammatory cytokines after RV14 infection. Previous reports have demonstrated that the reduced production of IL-6 by macrolides is associated with the inhibition of NF-*κ*B (Suzuki et al. 2001a, 2002). The inhibitory effects exerted by EM900 on NF-*κ*B activity that were observed in the present study are consistent with the findings of a previous study that showed that EM900 reduces IL-1*β*-induced NF-*κ*B activation in A549 cells (Otsu et al. 2011). NF-*κ*B increases the gene expression of ICAM-1 and pro-inflammatory cytokines (Zhu et al. 1996; Papi and Johnston 1999). Thus, EM900 might reduce ICAM-1 expression and cytokine production partially through the decrease in NF-*κ*B activation.

We used cycle threshold (Ct) values to measure RV RNA and ICAM-1 mRNA via RT-PCR. We observed that the Ct values of the *β*-actin mRNA in the HTE and HNE cells after infection did not differ from the values obtained prior to infection. These data suggest that RV infection did not affect *β*-actin mRNA levels in these cells. Therefore, the changes in RV14 RNA and ICAM-1 mRNA expression after RV14 infection might not be affected by changes in mRNA expression of *β*-actin, which was used as a housekeeping gene in the present study.

The endosomal pH is regulated by the vacuolar H^+^-ATPase (Mellman et al. 1986) and ion transport across Na^+^/H^+^ exchangers (Nass and Rao 1998). The macrolide bafilomycin acts as an inhibitor of the vacuolar H^+^-ATPase (Perez and Carrasco 1993) and increases the endosomal pH in HTE cells (Suzuki et al. 2001a). Erythromycin also increases the endosomal pH in HTE cells (Suzuki et al. 2002). Based on these findings, EM900 may have inhibited the vacuolar H^+^-ATPase in the cells in the present study.

Erythromycin reduced ICAM-1 mRNA expression prior to infection (Suzuki et al. 2002), whereas EM900 did not reduce the mRNA expression levels prior to infection in the present study. These finding suggest that erythromycin has more potent inhibitory effects on the expression of the RV14 receptor ICAM-1 compared to EM900. Further studies using cells from the same tracheae and nasal mucosal tissues are required to clarify the differences between the effects of erythromycin and EM900.

The inhibitory effects of EM900 are consistent with those of erythromycin, and the magnitude of the inhibitory effects of EM900 was similar to that of erythromycin in terms of the effects on RV14 titers, RV14 RNA replication, and the secretion of IL-1*β* and IL-6 (Suzuki et al. 2002). Therefore, the inhibitory effects of EM900 on RV replication and airway inflammation induced by RV infection may be similar to those induced by erythromycin. Furthermore, the long-term use of EM900 may not promote the growth of antibiotics-resistant bacteria because EM900 is a nonantibiotic macrolide (Sugawara et al. 2011). On the other hand, pretreatment of HTE and HNE cells with EM900 did not reduce ICAM-1 expression prior to RV14 infection in the present study, suggesting that EM900 may also have smaller potent effects on cell functions other than ICAM-1 expression compared with erythromycin. Gastrointestinal tract symptoms, including nausea and abdominal pain, were reported to be the most common adverse effects of macrolides, such as erythromycin (Yamaya et al. 2012). If EM900 has smaller potent effects on the gastrointenstinal motility through motilin-like activity (Yamaya et al. 2012) than erythromycin, the severity and frequency of the gastrointestinal symptoms may be reduced when COPD patients are treated with EM900. Therefore, EM900 could be a better drug to prevent COPD exacerbation compared to erythromycin, although EM900 had smaller potent inhibitory effects on the expression of ICAM-1 than erythromycin.

We previously reported that erythromycin pre treatment reduced RV14 titers by 1 − 1.5 logs (TCID_50_ units/mL) (Suzuki et al. 2002). Similarly, Jang et al. showed using A549 cells that clarithromycin pre treatment reduced RV16 titers by 1 − 1.5 logs (TCID_50_ units/mL) (Jang et al. 2006). Thus, the potency of the inhibitory effects of erythromycin and clarithromycin on RV replication seems to be small in in vitro studies. However, erythromycin and clarithromycin reduce the frequency of COPD exacerbation (Suzuki et al. 2001b; Seemungal et al. 2008; Yamaya et al. 2008), and erythromycin reduces the frequency of the common cold in COPD patients (Suzuki et al. 2001b). Although macrolides, including EM900, do not exert a potent inhibitory effect on RV replication, these findings suggest that the antiviral effects of macrolides are sufficient to exert therapeutic potential.

In the present study, pretreatment with EM900 did not reduce the viability of the attached cells or increase the number of detached cells or the concentrations of LDH in the supernatants. Sugawara et al. reported that a 100 μmol/L dose of EM900 induced cytotoxicity in the human monocytic leukemia cell line THP-1 (Sugawara et al. 2011). These findings suggest that 10 μmol/L of EM900 was not cytotoxic for the HTE and HNE cells and that the reduction in virus titers and RV14 RNA was not due to cytotoxicity.

There are some limitations in the evaluation of the results of this study. First, we demonstrated anti-RV effects of the nonantibiotic EM900 using human airway epithelial cells. In contrast, Abisheganaden et al. reported that the macrolide clarithromycin had little or no effect on the severity of common cold symptoms in experimental RV colds in a human clinical trial (Abisheganaden et al. 2000). In contrast, we previously reported that clarithromycin reduced fever duration in patients with influenza (Higashi et al. 2014), suggesting the anti-inflammatory effects of clarithromycin in influenza virus infection. However, further studies (either animal models or human studies) are required to confirm the potential value of EM900 for RV infection and the prevention of COPD exacerbation induced by RV infection.

Second, pretreatment of HTE and HNE cells with EM900 did not reduce ICAM-1 expression prior to RV14 infection. These results are consistent with those reported by Jang et al. that clarithromycin did not reduce ICAM-1 expression prior to infection but reduced RV replication and cytokine production in A549 cells (Jang et al. 2006). Although the mechanisms responsible for the inhibition of RV replication are still uncertain, the reduction of RV-induced increases in ICAM-1 expression by EM900 might have partially contributed to the reduction in RV replication and subsequent cytokine secretion observed in this study and the study by Jang et al. (Jang et al. 2006). However, in this study we did not detect an effect prior to RV infection, which was not very helpful for the prevention of COPD exacerbation. The epithelial cells were pretreated with EM900 for 72 h prior to RV14 infection in the present study. A longer time of pre treatment with EM900 may be more effective in the reduction of ICAM-1 and cytokine production, as reported for cytokine secretion from mice pretreated with clarithromycin (Terao et al. 2003). Because COPD patients are treated with macrolides for more than several months (Yamaya et al. 2012), long-term treatment with EM900 may be effective in preventing COPD exacerbation.

Third, we did not test whether the altered endosomal pH by EM900 inhibited RV entry. However, the specific vacuolar H^+^-ATPase inhibitor and macrolide bafilomycin A_1_ inhibited the entry of RV14 into the cytoplasm via endosomes in HeLa cells (Perez and Carrasco 1993). We also reported that bafilomycin A_1_ inhibited RV14 replication in HTE cells (Suzuki et al. 2001a). Because EM900 increased the endosomal pH similar to bafilomycin (Suzuki et al. 2001a), the increased endosomal pH may be one of the mechanisms contributing to the inhibition of RV14 infection in the present study.

## Conclusions

In conclusion, pretreatment with EM900 reduced RV14 replication and the production of pro-inflammatory cytokines in human tracheal and nasal epithelial cells in a manner similar to the inhibitory effects exerted by erythromycin in human tracheal epithelial cells. EM900 may inhibit RV infection by reducing ICAM-1 levels and the number of acidic endosomes, thereby modulating RV infection-associated airway inflammation.

## Conflicts of Interest

The authors have no conflicts of interest. Mutsuo Yamaya is a Professor in the Department of Advanced Preventive Medicine for Infectious Disease, Tohoku University Graduate School of Medicine. This department was funded by eight pharmaceutical companies, which are as follows: Kyorin Pharmaceutical Co. Ltd., Abott Japan, Co., Ltd., Taisho Toyama Pharmaceutical Co., Ltd., AstraZeneca Co. Ltd, Otsuka Pharmaceutical Co. Ltd., Teijin Pharma Co., Ltd., Toyama Chemical Co. Ltd., and Nippon Boehringer-Ingelheim Co., Ltd.
